# Specific Targeting of Plant and Apicomplexa Parasite Tubulin through Differential Screening Using In Silico and Assay-Based Approaches

**DOI:** 10.3390/ijms19103085

**Published:** 2018-10-09

**Authors:** Emmanuelle Soleilhac, Loraine Brillet-Guéguen, Véronique Roussel, Renaud Prudent, Bastien Touquet, Sheena Dass, Samia Aci-Sèche, Vinod Kasam, Caroline Barette, Anne Imberty, Vincent Breton, Marylin Vantard, Dragos Horvath, Cyrille Botté, Isabelle Tardieux, Sylvaine Roy, Eric Maréchal, Laurence Lafanechère

**Affiliations:** 1 Institut de Biosciences et Biotechnologies de Grenoble (BIG), Université Grenoble Alpes, CEA, INSERM, BGE U1038, CEA-Grenoble, 17 rue des Martyrs, 38000 Grenoble, France; emmanuelle.soleilhac@cea.fr (E.S.); loraine.gueguen@sb-roscoff.fr (L.B.-G.); veronique.roussel@uparc.fr (V.R.); caroline.barette@cea.fr (C.B.); sylvaine.roy@cea.fr (S.R.); 2 Sorbonne Université, CNRS, Integrative Biology of Marine Models (LBI2M), Station Biologique de Roscoff (SBR), 29680 Roscoff, France; 3 Laboratoire de Physiologie Cellulaire Végétale, Unité Mixte de Recherches 5168 CNRS, CEA, INRA, Institut de Biosciences et Biotechnologies de Grenoble (BIG), Université Grenoble Alpes, CEA-Grenoble, 17 rue des Martyrs, 38000 Grenoble, France; marylin.vantard@univ-grenoble-alpes.fr; 4 Institute for Advanced Biosciences (IAB), Team Regulation and Pharmacology of the Cytoskeleton, INSERM U1209, CNRS UMR5309, Université Grenoble Alpes, 38000 Grenoble, France; renaud_prudent@yahoo.fr; 5 Institute for Advanced Biosciences (IAB), Team Membrane and Cell Dynamics of Host Parasite Interactions, INSERM U1209, CNRS UMR5309, Université Grenoble Alpes, 38000 Grenoble, France; bastien.touquet@univ-grenoble-alpes.fr (B.T.); isabelle.tardieux@inserm.fr (I.T.); 6 Institute for Advanced Biosciences (IAB), Team ApicoLipid, CNRS UMR5309, Université Grenoble Alpes, INSERM U1209, 38000 Grenoble, France; Sheena.dass@univ-grenoble-alpes.fr (S.D.); cyrille.botte@gmail.com (C.B.); 7 Institut de Chimie Organique et Analytique (ICOA), UMR7311 CNRS-Université d’Orléans, Université d’Orléans, 45067 Orléans CEDEX 2, France; samia.aci-seche@univ-orleans.fr; 8 Laboratoire de Physique de Clermont, Université Clermont Auvergne, CNRS/IN2P3, UMR6533, 4 Avenue Blaise Pascal TSA 60026, CS 60026 63178 Aubière CEDEX, France; vinodkasam@gmail.com (V.K.); Vincent.Breton@clermont.in2p3.fr (V.B.); 9 Centre de Recherche sur les Macromolécules Végétales, Université Grenoble Alpes, CNRS, 38000 Grenoble, France; anne.imberty@cermav.cnrs.fr; 10 Grenoble Institut des Neurosciences; Inserm U1216; Université Grenoble Alpes, 38000 Grenoble, France; 11 Laboratoire de Chemoinformatique, UMR7140 CNRS—Université de Strasbourg, 4 rue Blaise Pascal, 67000 Strasbourg, France; dhorvath@unistra.fr

**Keywords:** Tubulin, dinitroanilines, plant cells, *Toxoplasma gondii*, *Plasmodium falciparum*, virtual screening, small molecules, cell-based assays

## Abstract

Dinitroanilines are chemical compounds with high selectivity for plant cell α-tubulin in which they promote microtubule depolymerization. They target α-tubulin regions that have diverged over evolution and show no effect on non-photosynthetic eukaryotes. Hence, they have been used as herbicides over decades. Interestingly, dinitroanilines proved active on microtubules of eukaryotes deriving from photosynthetic ancestors such as *Toxoplasma gondii* and *Plasmodium falciparum*, which are responsible for toxoplasmosis and malaria, respectively. By combining differential in silico screening of virtual chemical libraries on *Arabidopsis thaliana* and mammal tubulin structural models together with cell-based screening of chemical libraries, we have identified dinitroaniline related and non-related compounds. They inhibit plant, but not mammalian tubulin assembly in vitro, and accordingly arrest *A. thaliana* development. In addition, these compounds exhibit a moderate cytotoxic activity towards *T. gondii* and *P. falciparum*. These results highlight the potential of novel herbicidal scaffolds in the design of urgently needed anti-parasitic drugs.

## 1. Introduction

Microtubules (MTs) are hollow cylindrical polymers composed of α-β tubulin heterodimers. These highly dynamic assemblies organize the cytoplasm during interphase and form the mitotic spindle to segregate condensed chromosomes during mitosis. Microtubule organization shows a remarkable diversity in eukaryotes, with striking differences in clades deriving from photosynthetic ancestors. Animal microtubules are anchored on a structured microtubule-organizing center such as the centrosome, or in many differentiated animal cells they are arranged in non-centrosomal arrays that are non-radial [1]. In contrast, in vascular plant cells that lack a structurally defined microtubule-organizing center, interphase MTs are always organized into linear bundles that assume different configurations depending on the cell type [2,3]. In Apicomplexa single-celled eukaryotes, deriving from photosynthetic ancestors, although now lacking photosynthesis [4], such as *Toxoplasma gondii*, microtubule organization varies during the parasite life cycle. At the tachyzoite replicative stage, a corset of 22 evenly spaced sub-pellicular microtubules, anchored to the apical polar ring, critically directs the polarized and elongated shape of the zoite. In addition, this parasite builds an unusual microtubule-containing structure at the apical tip, which is named conoid [5]. In *Plasmodium falciparum*, a longitudinally oriented array of two–three sub-pellicular microtubules contributes to the shape and integrity of the parasite [6].

While α and β-tubulin are highly conserved proteins, the effects of microtubule-binding drugs vary in organisms belonging to distinct evolutionary groups. For example, plant tubulin and Apicomplexan tubulins have a much lower affinity for colchicine than animal tubulin [7]. In contrast, small synthetic molecules such as dinitroanilines (oryzalin, ethafluralin or trifluralin) bind specifically plant and Apicomplexa tubulins but not vertebrate or fungi ones [8,9,10,11]. Due to their selectivity towards plant tubulin, dinitroanilines have been used as herbicides for more than 40 years [7] and represent promising leads for the design of antiparasite drug candidates in particular in the case of *P. falciparum* and *T. gondii* [9,12].

Computational methods have provided evidences that the dinitroaniline binding site of *T. gondii* α-tubulin is located beneath the H1-S2 loop [13]. Such a location predicts the disruption of protofilament interactions in the microtubule lattice upon dinitroaniline binding.

Besides dinitroanilines and their derivatives, no chemical entities that selectively target tubulin of plants and parasites have yet been described. This is not the case for mammalian tubulin, which is the target of numerous diverse chemical compounds [14,15,16]. Therefore, to identify new chemical scaffolds that could be used as template for novel anti-parasitic drugs or herbicide, we have designed an integrated multi-step strategy. First, a differential in silico screen of small molecules from chemical libraries, docking to the α-tubulin dinitroaniline-binding site, was performed to select compounds that bind selectively to plant/parasite tubulins. The selected compounds were then screened on plant cells using a miniaturized assay. The compounds active on the plant cell MT cytoskeleton were further tested on plantlets viability and counter screened for their effect on the human cell cytoskeleton. A few residual molecules, active on the plant cell cytoskeleton and plantlets, but showing no detectable effect on human cells, were finally tested for their effect on in vitro tubulin assembly of plant versus mammalian tubulin. The combination of these approaches picked out three active molecules that are selectively active on plant tubulin. Remarkably, two of them are structurally different from dinitroanilines, and therefore represent novel scaffolds that serve as leads for the design of new generation herbicides. Additionally, we checked whether any of the retained candidates affect *T. gondii* and *P. falciparum* growth and survival within their relevant human host cells. One of these compounds showed a low but selective toxicity on the proliferative stages of *T. gondii* and *P. falciparum*, as compared to human cells, highlighting the usefulness of such a multidisciplinary approach to discover new classes of herbicidal molecules with potential anti-*Plasmodium* and anti-*Toxoplasma* properties.

## 2. Results

### 2.1. Determination of 3D Discriminating Conformations of P. falciparum α-Tubulin for In Silico Screening

**Figure 1:**
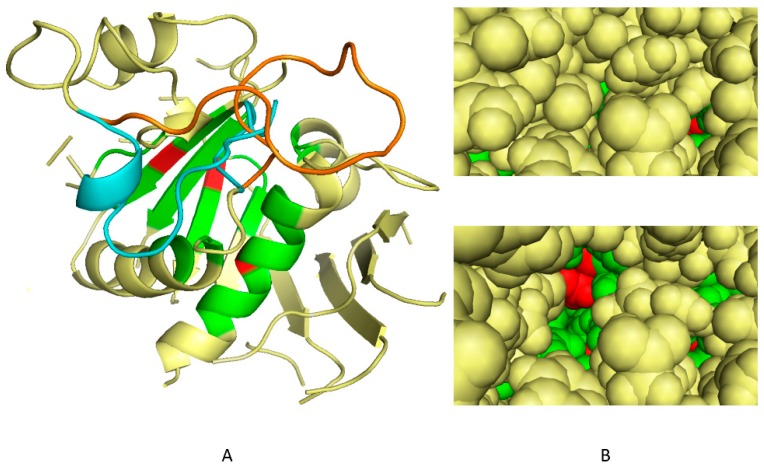
Determination of the best *P. falciparum*. α-tubulin conformations for in-silico screening. (**A**) Conformations of the H1-S2 loop (residues 35–60) of *P. falciparum* α-tubulin, showing the overlap of the initial homology model based on homology predictions to mammalian tubulin (cyan) and lowest-energy S4MPLE (Sampler for Multiple Protein-Ligand Entities)-generated open-site geometry model, which takes into account the dinitroaniline binding ability (orange). The residues Ser6, Ile235 and Leu167 in the dinitroaniline binding site of the *P. falciparum* tubulin that were found involved in interactions for every conformation and used as criteria for selection are coloured in red, and their immediate neighbourhood (within 4Å) is in green. The loop in the homology model—in cyan—is seen to block access to the site. When displaced to allow dinitroaniline binding ability the predicted new position (in orange) opens access to the three key residues. (**B**) View of the “red” key residues in a protein surface model, showing that they (and their immediate neighborhood, in green) are buried in the homology model (upper panel) but accessible in the sampled conformer model (lower panel).

α-Tubulin is a highly conserved protein (Figure S1). While tubulin structures have been obtained in multiple organisms and are available in the PDB database, the resolution level was not sufficient to be directly used as templates for the present differential in silico docking experiments. To perform a virtual screening on a domain conserved only in the photosynthetic lineage, in broad sense, we first selected a representative tubulin structural model in an Apicomplexa, well known to be non-photosynthetic today but deriving initially from a photosynthetic ancestor [17,18]. The sequence of *P. falciparum* α-tubulin (Uniprot accession: CAA34101) was thus used, focusing on regions conserved in plants. *P. falciparum* α-tubulin structure was determined by homology modeling [19] using bovine (AAX09051) and porcine (P02550) α-tubulin crystal structures as templates (Figure S2, step1). In the predicted structure, the H1-S2 loop (residues 35–60) locked the oryzalin-binding site, preventing molecules from penetrating inside. An early version of the conformational sampling tool S4MPLE [20] specifically operating on the torsional degrees of freedom only [21] was used to explore alternative putative poses of that loop. Main chains and side chains of the loop aminoacids, as well as side chains of residues putatively in contact with loop residues were declared mobile, while freezing the rest of the protein to its initial geometry. In order to sample a protein loop anchored to a rigid protein core at both ends, S4MPLE (Sampler for Multiple Protein-Ligand Entities, an algorithm designed for the conformational sampling of small molecules and in-silico docking experiments) needs an input of a user-chosen identifier of an existing main chain bond (here, the N-C α of the loop-central aminoacid, i.e., between residues 28 and 47), which will be formally considered as “broken”. This allows free movement of the formally disjoined loop moieties in S4MPLE, while accounting for the complete molecular Hamiltonian (based, in that version of S4MPLE, on the CVFF force field [22]), i.e., including the concerned bond stretching and associated valence angle bending terms. This “trick” ensures a full sampling of possible loop geometries, while selecting only those that are properly closing the artificial "gap" and providing consistent geometries for the covalent elements. Since all other bond length and valence angle values were not subjected to changes (and remained set to their input values), the chirality of the C α involved in the formally broken bond was implicitly conserved. Several independent simulations of the system were run, using a genetic algorithm-based sampling strategy, for 1,000 generations each, until it was observed that, for 10 successive simulations, no absolutely lower energy value could be attained. We selected 100 conformers among the more stable ones according to a criterion of diversity, measured mainly on torsional axes (Figure S2, step 2). As shown in Figure 1, these conformers (Figure 1B, lower panel) present a well-formed “dockable” cleft as compared to the initial homology-modeled geometry (Figure 1B, upper panel).

To select the more likely conformations, we then docked on these 100 conformers a reference set of 37 molecules comprising 10 dinitroaniline derivatives known to target the dinitroaniline-binding site [13] and 27 compounds never reported for binding tubulin, to our knowledge. By ranking the conformers according to their ability to discriminate between the active/non active molecules we could select the five more likely conformations of the H1-S2 loop (Figure S2, step 3).

These five conformations as well as the mammalian and the original *Plasmodium* tubulin conformations were the final targets for the in silico differential screening.

### 2.2. In Silico Selection of Compounds that Bind to the Dinitroaniline Site of α-Tubulin from the Photosynthetic Lineage

Using the five most promising conformations, a virtual library of more than 300,000 chemical compounds was screened for its ability to dock into the identified cleft. We first analyzed the FlexX scores in the whole docked database to evaluate the possibility to rank conformations based on these scores. This analysis showed that the active compounds previously reported in the literature were close to the average value in the score distribution and not among the best scores. The use of FlexX scores was thus considered as not discriminant enough to select molecules. Post-processing of docking outputs through analyses of the three dimensional protein-ligand binding interactions has been reported to be a powerful alternative strategy [23,24,25]. A thorough analysis of the interaction data obtained for known active compounds on the 100 conformers described above revealed that numerous residues involved in the interactions were located at the pocket entrance, whereas the residues located in the depth of the pocket were more rarely involved. Among them, three residues (Ser6, Ile235 and Leu167, Figure S1) were involved in interactions for every conformation (Figure 1B). We decided to use them as a criterion for selection.

Based on the protein molecule interactions, 3,023 molecules that, in one or more of the five “open” protein conformations, had one or more docking poses in interaction with Ser6, Ile235 or Leu167 were selected. We checked that, among these compounds, none had been selected for their effect on mammalian cell microtubules, in previous screening campaigns [15,16]. This was never the case, therefore validating the proposed specificity of the binding pocket for the photosynthetic lineage. Among these 3,023 compounds, 82 molecules were readily available in the laboratory and therefore picked for further in vitro analysis.

### 2.3. Selection of Compounds Active only on Plant Cell Microtubules

The selected compounds were tested for their effect on tobacco BY-2 cell microtubules using immunofluorescence. Image-based screening is frequently performed on adherent mammalian cells [16,26] but has never yet been achieved on plant cells. We thus developed an optimized immunostaining procedure in 96-well plates, as described in the Material and Methods section and captured images using an automated fluorescent microscope.

**Figure 2:**
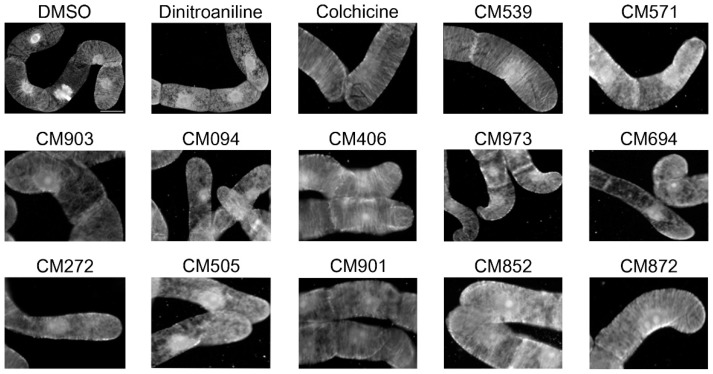
Effect of selected compounds on plant cell microtubule network organization. BY2 cells were incubated for 2 h with 0.25% DMSO (vehicle control); 25 µM dinitroaniline, 2 µM colchicine and 50 µM of the indicated compounds. Cells were then processed for tubulin immunofluorescence as described in Material and Methods. CM539 is an example of a compound that was found inactive. Bar = 20 µm.

**Figure 3:**
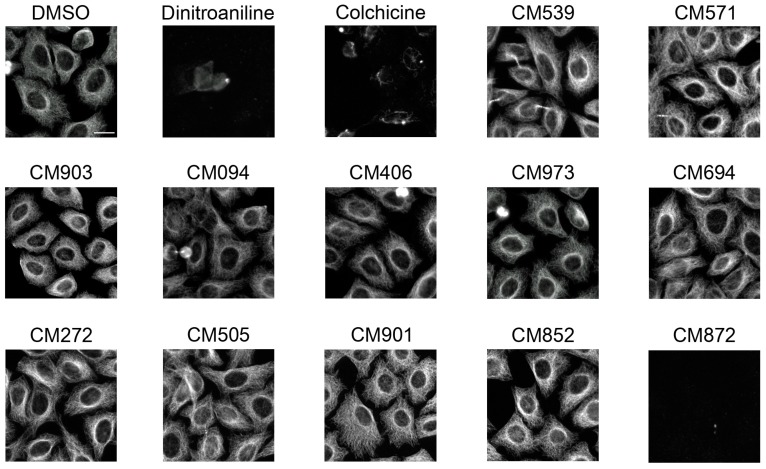
Effect of selected compounds on mammalian cell microtubule network organization. HeLa cells were incubated for 2 h with 0.25% DMSO (vehicle control); 25 µM dinitroaniline, 2 µM colchicine and 50 µM of the indicated compounds. Cells were then processed for tubulin immunofluorescence as described in Material and Methods. Bar = 20 µm.

The effect of a two-hour incubation with each of the 82 molecules (50 µM) was compared to those of dinitroaniline (25 µM) that depolymerizes plant MTs, and colchicine (2 µM) that has low affinity for the plant tubulin. As expected, MT depolymerization was observed upon dinitroaniline treatment whereas colchicine did not affect the plant-cell microtubule network (Figure 2). Eleven compounds, out of the 82 compounds tested, were found to have an effect on cortical MTs (Figure 2). This indicates that these compounds could be able to cross the BY-2 cell membrane to impact cellular microtubules. Their effect on mammalian cell microtubules was then tested by immunofluorescence using HeLa cells. When applied at concentrations ranging from 3 to 50 µM for 2 h at 37 °C none of the compound, except compound CM872, induced detectable effects on microtubules. Compound CM872 showed a toxic effect that was detected at the higher concentration tested only, i.e., 50 µM (Figure 3). Overall, 10 compounds were found to be active only on BY-2 cell MTs with no detectable effect on HeLa cell MTs.

In order to know if these compounds could penetrate into a whole plant organism and be active, they were finally tested for their effect on *Arabidopsis thaliana* growth. As shown in supplementary Figure 3, three compounds, i.e., compounds CM571, CM094, and CM852 inhibited plant growth. In addition, severe symptoms of chlorosis could be detected for compounds CM571 and CM852, indicating that they circulated in plant vascular tissues and induced a systemic response (Figure S3).

### 2.4. Selected Compounds Target Plant but not Mammalian Tubulin

**Figure 4:**
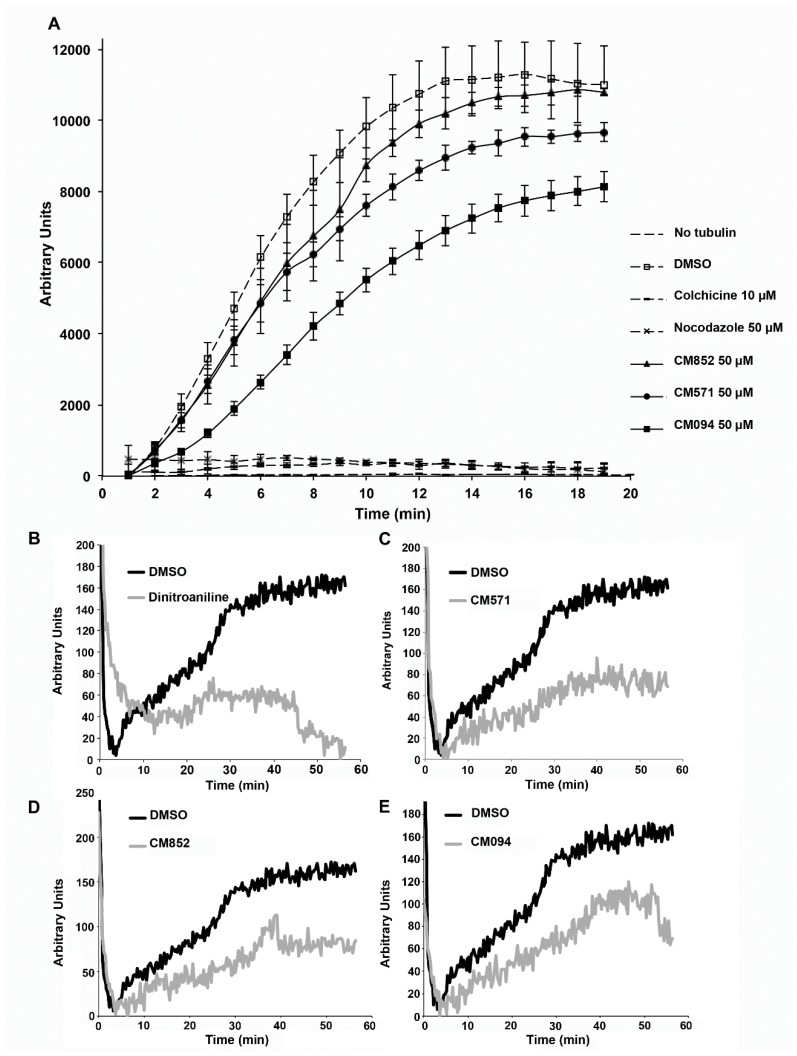
Comparison of the effects of CM094, CM571 and CM852 on mammalian and plant microtubule polymerization in vitro. (**A**) Pure bovine brain tubulin polymerization assay. Tubulin was allowed to polymerize at 37 °C. Fluorescence of DAPI bound to microtubules was measured to monitor microtubule polymerization, as described in the Material and Methods section. Experiments were performed in triplicate, in the presence of the indicated compounds. Results are presented as mean ± standard error of the mean (SEM). Effect of dinitroaniline (**B**), CM571 (**C**), CM852 (**D**), and CM094 (**E**) on soybean tubulin assembly. Recombinant soybean tubulin was polymerized as described in the Material and Methods section, in the presence of DMSO (control) or the indicated compounds.

We next focused on these three compounds and evaluated at the molecular level whether they targeted plant tubulin but not mammalian tubulin. We thus compared their effect, at concentrations ranging from 0.1 to 50 µM on the in vitro assembly of tubulin purified from soybean or bovine brain. In contrast to colchicine and nocodazole, CM571, and CM852 even at high concentration (50 µM) did not show any significant effect on the assembly kinetics of bovine brain tubulin, in agreement with the lack of detectable changes in the cellular microtubule network of intact HeLa cells (Figure 4A). Of note CM094 induced a slight depolymerizing effect at 50 µM but had no effect at lower concentrations [27] While soybean tubulin was less assembly competent, we observed tubulin polymerization reaction under control conditions that was no longer detected in presence of dinitroaniline (Figure 4B). All three compounds impaired soybean tubulin assembly (Figure 4C–E). After dose-response analysis [27], the drug inhibitory profile is estimated as follows: dinitroaniline > CM852 > CM571 > CM094.

**Figure 5:**
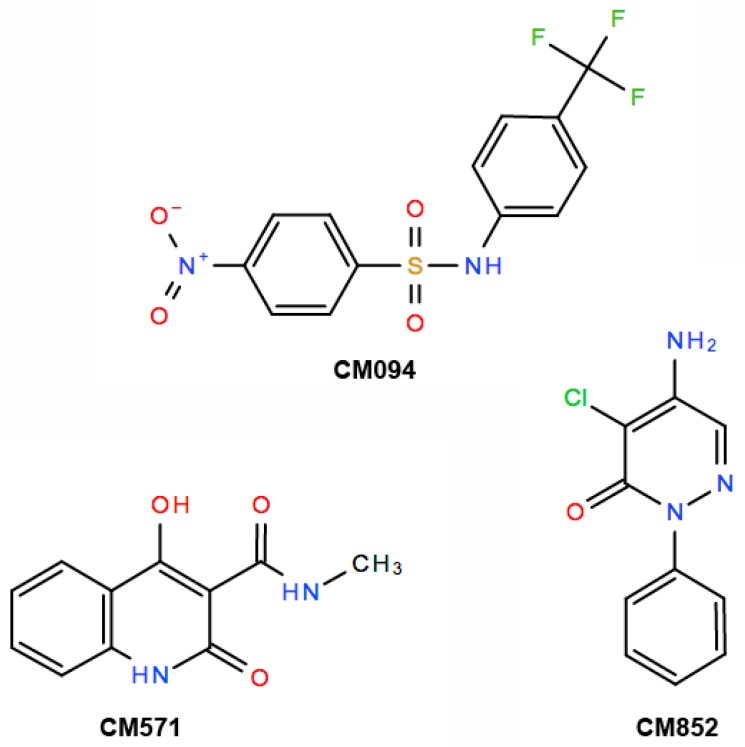
Structure of the selected compounds.

The comparison of the chemical structure of the compounds (Figure 5) showed that the three compounds belong to different classes of chemicals. No biological effects of CM571 have so far been described. Interestingly, CM094 shares common features with dinitroaniline, namely a nitro-phenyl-sulfonamide moiety, which may mediate the observed effect on plant tubulin assembly. Likewise, CM852 is a pyridasinone (n-chloridazon) that is currently used as a selective systemic herbicide [28]. Among other properties such as DNA intercalation and interference with the synthesis of fatty acids [29], n-chloridazon is primarily known to inhibit photosynthesis and thus to induce chlorosis, a symptom we observed in *A. thaliana* plantlets.

We concluded from these experiments that CM852, CM571, and CM094, originally selected by a virtual screen to bind to the dinitroaniline binding site of *P. falciparum* tubulin, were indeed able to target plant tubulin with no detectable effect on mammalian tubulin.

### 2.5. Analysis of the Effects of the Compounds on Apicomplexan Parasites

**Figure 6:**
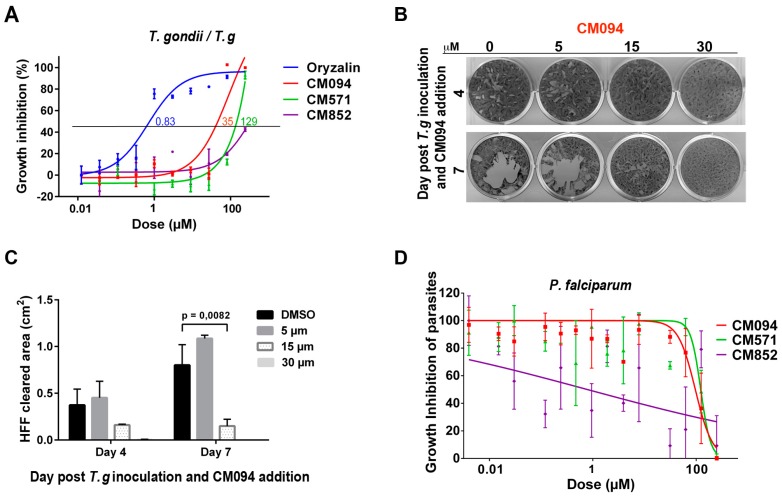
Effect of selected compounds on *T. gondii* and *P. falciparum*. (**A**) The effect of the compounds on *T. gondii* replication within human fibroblasts was tested. The graph presents *T. gondii* growth inhibition curves for each treatment at concentrations ranging from 0.01 to 100 µM, in triplicates. The quantity of individual parasites within intracellular vacuoles exposed or not to the drug for 30 h post-invasion was automatically scored. The calculated IC_50_ is indicated for each compound. (**B**) Plaque assays showing at 4 and 7 days the expansion of HFF cleared zones due to successive rounds of lytic cycle that were initiated by a single parasite. HFF monolayers plated on a 6-well plate were inoculated with 200 invasive tachyzoites per well for 1 h. The non-invading tachyzoites were washed away and cells were incubated with medium containing the vehicle (DMSO, 0) or different concentrations of CM094. After fixation and Crystal violet staining, cells were air dried and scanned before image processing and plaque area measurement. At day 7, post-inoculation, a highly significant reduction of parasite expansion was observed for the cultures exposed to 15 µM of CM094 and no plaque was detected at 30 µM while the HFF monolayer was well preserved. (**C**) Histograms showing the cumulated area of HFF cleared zones. *p* value = 0.0082. (**D**) The effect of selected compounds on *P. falciparum* intra-erythrocytic growth was tested. The growth of parasites within human red blood cells is determined using a classical SYBR Green assay followed by quantification of parasite DNA fluorescence. The graph presents growth curves for concentrations of each compounds ranging from 0 to 250 µM, in triplicates.

Due to the sensitivity of *T. gondii* to dinitroanilines that has already been reported with IC50 values for commercially available dinitroanilines ranging from 45 nM to 6.7 μM, we tested whether the selected compounds could impact *T. gondii* and *P. falciparum* intracellular development within their respective host cells. Taking into account that the subpellicular MTs of extracellular parasites are non-dynamic and notoriously insensitive to MT pharmacological disruptors, we had to perform the assays on intracellular multiplying parasites. This experimental requirement implies that the compounds of interest could access not only the host cell cytosol, but also pass the membrane of the parasitophorous vacuoles, within which the tachyzoite multiplies and ultimately reach the parasite cytosol. Using fluorescent *T. gondii* tachyzoite and High Content Imaging that allowed quantitative detection of individual progeny within intracellular parasitophorous vacuoles post infection, we found, as expected, that the dinitroaniline analog oryzalin was highly and selectively potent on parasites, with an IC_50_ of 0.8 µM (Figure 6A, blue line). Oryzalin IC_50_ reached 99 µM for the human host fibroblasts (HFF). Accordingly the selectivity index, defined as the ratio of IC_50_ human fibroblasts/ IC_50_ *T. gondii*, reached 123.7. We found that compounds CM094 and CM571 moderately impacted parasite growth with an IC50 of 35 and 130 µM, respectively (Figure 6A, red and green lines), whereas compound CM852 had no detectable effect (Figure 6A, purple line). CM094 and CM571 showed no significant effect on human fibroblasts, with IC50 of 240 and 237 µM, respectively [30], resulting, however, in lower selectivity indexes than oryzalin (i.e., 6.8 and 1.8, respectively). The compound effectiveness towards its target, the dynamic MTs, is significantly challenged by the need of successive translocations across three biochemically different membranes. Thus, we next tested for a longer time period the activity of the most potent compound on *T. gondii* tachyzoite proliferation, defined with the IC_50_, i.e., CM094. To this end, we incubated 200 invasive tachyzoites on HFF monolayer in a six-well plate (9.61 cm^2^/well) and added the drug immediately after one hour of invasion. The time window was adjusted to allow a single parasite to multiply and the progeny to undergo several rounds of infection or “lytic cycles”. Under these conditions, we observed a significant difference in the size of HFF cleared areas between cells exposed to CM094 or to the vehicle alone. The effect was already observed after four days and drastically increased with time (Figure 6B,C). Assuming a conserved rate of infection between each sample, these assays document a significant loss of parasite fitness in presence of 15 µM of the compound and attest to the integrity of the HFF monolayer under drug treatment.

We then decided to investigate whether the compounds had an effect on the proliferation of *P. falciparum* (the most lethal agent of human malaria) during the intra-erythrocytic life stages, which are the symptomatic stages of the malaria infection. We thus incubated a tightly synchronized 3D7 ring wild-type population (i.e., initial intracellular developmental stage of the blood phase, 0.5% parasitaemia) in a 96-well plate (2% hematocrit) in the presence of different concentrations of the compounds ranging from 4 nM to 250 µM. Parasites were left in culture to undergo the intra-erythrocytic life cycle of 48h, which usually allows the 3D7 strain to increase its population of about 10-fold. Parasitaemia after drug treatment was determined by a typical SYBR Green assay [31]. Compound CM852 (Figure 6D, purple line) showed highly variable effects on the parasite growth whereas both compounds CM094 (Figure 6D, red line) and CM571 (Figure 6D, green line) slowed down the intracellular proliferation of *P. falciparum* at high concentrations. The IC_50_ values of CM094 and CM571 were determined to be 94.7 and 122.4 µM, respectively. Notably, no cytotoxic effect could be observed on the human host erythrocytes [32], thus suggesting a specific effect on the parasite itself.

These results identified CM094, the compound most closely related to dinitroanilines as the only one to significantly impact the *T. gondii* tachyzoite lytic cycle. Both CM094 and CM571 were found to impact the trophozoite intra-erythrocytic development in *P. falciparum*.

## 3. Discussion

By integrating in silico and assay-based approaches we were able to explore the diversity of chemical space to find agents able to bind to the dinitroaniline tubulin-binding site. These compounds were assayed on evolutionary close and distant organisms, which allowed the identification of drugs with new chemical scaffolds that target plants and to a moderate extent, Apicomplexa parasites, while sparing animal cells.

Eleven compounds, out of the 82 in vitro-assayed compounds, were found active on the plant BY-2 microtubule cytoskeleton. This unusual high ratio points out the efficiency of the docking step strategy likely resulting from the combination of the following factors. First, a great number of ligands (300,000) have been docked thanks to the grid computational power, increasing the probability to select active compounds. Second, the S4MPLE algorithm and the access to the grid allowed an exploration of the possible conformations of the targeted site. Using a pre-docking step with known active and putative inactive ligands allowed the selection of the five most probable conformations. The docking on these five conformations was thus conducted on judiciously restricted but flexible targets. Finally, the docking poses and the important amount of interaction data have been scrutinized both manually and with homemade scripts. This in-depth analysis allowed the definition of a subset of criteria that were subsequently proven to be relevant.

While numerous compounds targeting mammalian tubulin are described, much less is known regarding drugs acting on plant tubulin. One reason is that the systematic research of compounds that selectively affect plant tubulin assembly is hampered by the poor availability of purified assembly competent plant tubulin. High content screening methods, based on the visualization of the compound effect on plant cell microtubule networks, could represent a convenient alternative. Here, we did not use computer-based methods for the analysis of the high content screening experiments, which allowed the evaluation of compound effects on BY-2 cell microtubules, but care has been taken to develop a standardized protocol that could be readily automated. BY-2 cells have proven to be a good cell model for this screening but other plant cells could also be valuably used in similar screens. We anticipate that high throughput screens of the effect of large sets of compounds on plant proteins in the cellular context will be easily implemented and benefit of our methodological developments. Such a method could also be used in a chemical genetics approach to identify chemically targetable microtubule regulators [33,34].

Biochemical experiments confirmed that plant tubulin is the in vitro target of the three selected compounds and that their binding to tubulin is likely responsible for MT depolymerization. Their toxic effect on plant cells and their effect on plant growth can therefore most probably be attributed to their effect on tubulin dynamics. It has been shown, however, that oryzalin also induces changes in the morphology of the endoplasmic reticulum (ER) and Golgi apparatus, which could contribute to its herbicide properties [35]. Such additional effects could also be responsible of the observed herbicide activity of the compounds we have selected, especially CM094, which shares some structural similarities with dinitroaniline.

Interestingly compound CM852 (pyrazon [5-amino-4-chloro-2-phenyl-3(2*H*)-pyridazinone]) is already described as an herbicide that works by blocking electron transport in photosystem II in green plants, thereby inhibiting photosynthesis. Its inhibitory effect on tubulin assembly, uncovered in this work, is likely reinforcing its toxic effect on plants. Available toxicity data on this compound indicate that it is of low toxicity without highly specific responses in mammals. This study confirms the selectivity of CM852 and the other two selected compounds for plant cells since none of them affected human fibroblastic and epithelial cell viability, in full consistency with the fact that they do not target mammalian tubulin. Moreover, the absence of their toxicity indicates that these compounds are not highly reactive and that they do not target proteins important for cell viability. Notably, since at least the CM094 compound has proven some activity on *T. gondii* intracellular growth and to a lesser extent on *P. falciparum*. This argues that CM094 can cross multiple biological barriers, and remain active. Remarkably, to target MTs dynamics that occur during apicomplexan zoites multiplication (i.e., *T. gondii* tachyzoites and *P. falciparum* trophozoite), the drug has to successfully translocate across the host cell plasma membrane, the membrane of the vacuole which houses the replicating tachyzoites and the plasma membrane of the tachyzoite itself. Whether the anti-tachyzoite MT activity of CM094 is reduced due the multistep obligate trafficking of the drug to reach its target once delivered into the culture medium remains to be evaluated. Previous work on the effect of dinitroanilines on *P. falciparum* corroborate our results showing a moderate activity on *P. falciparum*, but a very low mammalian cytotoxicity of the compounds [36]. This work also concluded that due to their hydrophobic nature, dinitroaniline derivatives largely accumulate in the parasite membranes. This reduces the amount of molecules having access to their microtubular target, likely explaining the modest effect observed on *P. falciparum*. Synthesis of chemical derivatives of CM094 may overcome this limitation and gain in anti-malarial activity.

The development of new herbicides, with no negative impact on humans and on the wild fauna, would have important consequences at the ecological and economical levels. While the compounds we have described act in the micromolar range, their chemical structure is simple. These molecules thus provide a useful platform for compound optimization. They also exhibit a selective effect on *T. gondii* and *P. falciparum* proliferation and could thus represent alternative scaffolds to the dinitroaniline analogs, in particular the meta-amino derivatives that have been already characterized as potent anti-*T. gondii* reagents [37]. Considering the urgent need for the development of therapeutic agents against malaria and other parasitic diseases, uncovering new scaffolds as leads for the future design of selective anti-parasitic drugs remains a priority.

## 4. Materials and Methods

### 4.1. Chemical Reagents, Recombinant and Purified Protein

Reagents used include DMSO (Sigma, D5879, Saint-Quentin Fallavier, France); oryzalin (Sigma, 36182); paclitaxel (Sigma, T1912); colchicine (Sigma, C9754); nocodazole (Sigma, M1404); phosphate buffer saline (PBS, Sigma P4417); foetal bovine serum (FBS, Sigma); formaldehyde (Sigma, F1635); glutaraldehyde (Polysciences, Inc, Warrington, PA, USA); Tween 20 (Sigma, P9416); sodium azide (Merck, Lyon, France, 6688); piperazine-*N*,*N*′-bis(2-ethanesulfonic acid (PIPES, Sigma, P6757); ethylene glycol-bis(β-aminoethyl ether)-*N*,*N*,*N*′,*N*′-tetraacetic acid (EGTA, Sigma, E4378); MgCl_2_ (Sigma, M1028); Triton X100 (Sigma, T8787); Glycerol (Sigma); phenylmethylsulfonyl fluoride (PMSF, Sigma); 2-(*N*-morpholino)ethanesulfonic acid (MES, Sigma, M3671); 4′,6-diamidino-2-phénylindole (DAPI, Sigma, D8417); CaCl_2_ (Sigma, C5670); mannitol (Sigma, M4125); pectolyase Y23 (Seishin Pharmaceutical, Tokyo, Japan); macerozyme R-10 (Serva, Heidelberg, Germany); caylase 345 (Cayla, Toulouse, France); Hoechst 33342 reagent (Sigma, B2261); protease cocktail inhibitors (Sigma, P8340); phosphatase cocktail inhibitors (Sigma, P5726); bovine serum albumin (BSA, Sigma, A3059); normal goat serum (Interchim, Montluçon, France); 3-[4,5-Dimethylthiazol-2-yl]-2,5-diphenyltetrazolium bromide (MTT); Thiazolyl blue (Sigma, M5655). Tubulin was prepared from fresh bovine brains as described in Paturle-Lafanechère et al. [38]. Soybean tubulin was purchased from Cytoskeleton, Inc. (Denver, CO, USA). Compounds screened were from ChemBridge Corporation (San Diego, CA, USA).

### 4.2. Antibodies

The α-tubulin antibody used was from clone α3a [39]. Anti-mouse IgG secondary antibodies conjugated with cyanine 3 were from Jackson ImmunoResearch Laboratories (Cambridgeshire, UK).

### 4.3. Mammalian, Plant and Apicomplexan Cell Lines

Mammalian cells: HeLa cells were originally purchased from the American Type Culture Collection (ATCC, Middlesex, UK), and maintained in Roswell Park Memorial Institute 1640 (RPMI-1640) medium supplemented with 10% (*v*/*v*) FBS and 1% penicillin/streptomycin, instead of DMEM, because the assayed compounds were more soluble in RPMI. MES-SA cells and MES-SA-DX5, from ATCC, were first grown in McCoy’s 5A (ATCC) with 10% (*v*/*v*) FBS and 1% penicillin/streptomycin and further adapted to grow in RPMI-1640 with 10% (*v*/*v*) FBS and antibiotics. These mammalian cell lines were maintained at 37 °C with 5% CO_2_ and 3% O_2_. Cells were treated with the compounds for 2 h unless otherwise stated. Plant cells: Tobacco BY-2 cells (*Nicotiana tabacum* L. Bright-Yellow 2, Riken) were grown in suspension according to Nagata et al. [40]. Primary human foreskin fibroblasts (ATCC CRL-1634) were seeded at about 70–80% of confluence in P96 and P6 well plates in high glucose Dulbecco’s Modified Eagle’s Medium (DMEM), supplemented with 10% (*v*/*v*) heat-inactivated fetal bovine serum (FBS) and 10 mM HEPES pH 7.0 (i.e. complete medium) and used 48–72 h later when confluent. Apicomplexan cell lines: The *T. gondii* RH strain expressing cytosolic GFP was maintained by serial passage in Human Foreskin Fibroblats (HFF) monolayers maintained in complete medium and as previously described [41]. *P. falciparum* 3D7 cultures were grown, as previously described [42] in 2% hematocrit (obtained from Etablissement du Sang Français, Grenoble, France) in RPMI medium (Thermo Fisher Scientific, Waltham, MA, USA) complemented with 10% Albumax (Thermo Fisher Scientific), Gentamycin (Merck Sigma Aldrich, Lyon, France) and hypoxanthine at 37 °C with a beta gas mix (1% O_2_, 5% CO_2_, 96% N_2_).

### 4.4. In Silico Screening on Alpha-Tubulin 3D-Model: Virtual Library of Compounds, Molecular Docking and Processing Methods

The dinitroaniline binding site of α-tubulin has been described in previous studies, located beneath the H1-S2 loop [13]. A sub-library of compounds, comprising 307,802 molecules available at ChemBridge Corporation was retrieved from the ZINC database (http://zinc15.docking.org/) in mol2 format with the hydrogen atoms and the atomic partial charges. The virtual library was massively docked on the EGEE (Enabling Grids for E-sciencE) European grid infrastructure [43] with the FlexX software [44] at the level of the putative dinitroaniline/oryzalin site. FlexX docking results contained several groups of data: (1) for each compound docked on each protein conformation, the 10 best poses were returned in mol2-format output files; (2) associated to each pose, the software returned also files giving the FlexX score value and the interactions between the protein residues and the ligand. The amount of information associated with 10 poses of 307,802 molecules docked on 7 active sites represented about 300 GB of textual data. To process this huge amount of data, analysis scripts were developed in Unix Shell, Unix Awk, or Perl languages; in order to visualize and sort the interesting values, the R programming language and the Kyplot package were used for statistics. To visualize, align ligands and to inspect, manually, their interactions with the dinitroaniline/oryzalin active site, the following software were used: Visual molecular dynamics VMD [45] Chimera [46], Ligplot [47]), MOE (Molecular Operating Environment, Chemical Computing Group–1010 Sherbrooke Street West, Montreal, Canada H3A2R7) and Hermes, the graphical interface of Gold [48].

### 4.5. Microtubule-Interference Assay in BY-2 Tobacco Cells

Samples of 90 µL of BY-2 cells, grown for 2.5 to 6.5 days, were transferred into 96-well polystyrene plates (Masterblock 2 mL, Greiner, Courtaboeuf, France) and supplemented with 10 µL of the chemical compounds (final concentration 50 μM). The cells were then incubated for 2 h at 27°C in the dark and under gentle agitation. Cells were further fixed by the addition in each well of 900 µL of MBS buffer (50 mM PIPES, 5 mM EGTA, 1 mM MgCl_2_, 2% Glycerol, pH 6.9) containing 3.7% formaldehyde, 1% glutaraldehyde, 1% DMSO, 0.5% Triton X-100 and 200 µM phenylmethane sulfonyl fluoride (PMSF), for 15 min under gentle agitation. The fixation buffer was then removed after sedimentation of the cells under centrifugation (2 min, 100× *g*) and replaced by 500 µL of MBS buffer for 10 min. This washing step was repeated 4 times. A volume of 50 µL of cell suspension was then transferred from Masterblock plates to Microclear 96-well flat bottom polypropylene plates (Greiner Bio-One #655090) coated with poly-d-Lysine. After a 15 min sedimentation period, cell walls were permeabilized for 2 min with 25 mM MES, 8 mM CaCl_2_, 600 mM Mannitol, 0.02% Pectolyase, 0.1% Macerozyme, 0.3% Caylase at pH 5.6, at room temperature. They were then washed with 50 µL of MBS buffer, and incubated with 5% of normal goat serum for 2 h. After 5 washes with MBS buffer, 45 µL of anti-tubulin antibodies at 1/5000 dilution were added in each well and incubated overnight. After 5 washes with MBS buffer, cells were incubated with Cy3 secondary antibodies (dilution 1/2000) and Hoechst (dilution 1/1000) for nuclei staining for 2 h. Finally, after 5 washes in PBS, the BY-2 cells were stocked in 50 µL of PBS/glycerol (50/50) at 4 °C before automated imaging. Compounds found to be active were systematically tested again from freshly made solutions.

### 4.6. Microtubule-Interference Assay in Hela Cells

The effect of compounds on microtubules was assayed in HeLa cells in 96 well-microplates using immunofluorescence, after permeabilization, and fixation of the cells, as previously described [16].

### 4.7. Drug Effect on Toxoplasma gondii and Plasmodium falciparum Parasite In Vitro

The activity of compounds against *T. gondii* in vitro was tested by (i) automatic scoring of GFP-expressing parasites within intracellular vacuoles over 30 h post invasion and in presence of a wide range of compound concentration. These assays allowed assessing the growth potential over about 4 to 5 replication cycles (ii) analysis of clear zones developing in a host fibroblast monolayer over 4 and 7 days as a result of the cell lysis induced by tachyzoite multiplication following infection by a single parasite. The so-called comparative plaque assay therefore revealed the additive defects on growth, motile, invasive and egress capabilities caused by the compound under study over numerous cycles of infection. To first monitor parasite multiplication over the 30 h period, we applied high content screening using fluorescence microscopy on 96 well plates with 20 fields of acquisition for each well and performed in triplicates for each condition. Images were further processed with Scan^R software (Olympus Life Science, Rungis, France). In the plaque assay, area of clear zones in the HFF monolayer were defined by image segmentation and measured following ethanol fixation and Crystal violet (0.05% in distilled water) staining with ImageJ software (https://imagej.nih.gov/ij/, [49]) and for all assays the datasets were analyzed using GraphPad Prism 6 software (La Jolla, CA, USA) and organized using Adobe Photoshop CS6 software (Paris, France).

Activity of compounds on *P. falciparum* intra-erythocytic life stages was assayed on tightly synchronized cultures of 3D7 ring culture using a classical SYBR Green assay, as previously described [31]. Briefly, populations of infected red blood cells with 0.5% 3D7 *P. falciparum* rings were allowed to grow in culture medium containing various concentrations of compounds (ranging from 4 nM to 250 µM) for an entire intra-erythrocytic life cycle of 48 h in 96-well plates (Thermo Fischer Scientific). Parasites (i.e., infected red blood cells) were quantified using SYBR Green II (Merck Sigma Aldrich), which gives a green fluorescence upon its contact with *P. falciparum* DNA (erythrocyte lacking nuclei and DNA, fluorescence is specific to the parasite presence). Fluorescence quantification was measured using a CLARIOstar 96-well-plate reader (BMG Labtech, Champigny s/Marne, France) and data was analyzed using GraphPad Prism 6 software.

### 4.8. Automated Imaging

Automated imaging of cells seeded in 96-well clear-bottom plate (Greiner, #655090) was performed on INCell Analyzer 1000 (GE-Healthcare, Cardiff, UK) using an 20x air objective. Eight images/well were acquired in the center of the well. Excitation and emission filters pairs used for nuclei, A488 and Cy3 staining were 360 nm/460 nm, 475 nm/535 nm and 535 nm/620 nm, respectively. Acquisition parameters such as exposure time and Z-plane focus were specifically fixed for each immunofluorescence assay.

### 4.9. Arabidopsis Thaliana Growth Assay

Arabidopsis seeds were obtained from Lehle seeds Inc. (Round Rock, TX, USA). Plants were grown on solid agar medium containing Murashige and Skoog (Caisson Labs, Smithfield, USA; MSP09) growth medium complemented with molecules supplied as described, in 24-well microplates, for 3 days in a humid chamber. Plants were then transferred in a growth chamber for 2 weeks at 20 °C with white light (100 mmol m^−2^·s^−1^) and a 16/8-h photoperiod. Observations were realized 7 days after transfer.

### 4.10. Tubulin Polymerization Assay

Microtubule polymerization assay was described in Prudent et al. [34], with final tubulin concentration of 25 µM for bovine tubulin or soybean tubulin.

### 4.11. Analysis of Cell Viability Using 3-(4,5-Dimethylthiazol-2-yl)-2,5-diphenyl-tetrazolium bromide (MTT)

Cytotoxicity was evaluated with a 3-(4,5-dimethylthiazol-2-yl)-2,5-diphenyl-tetrazolium bromide (MTT) colorimetric assay, performed in 96-well microplates as described in Martinez et al. [16].

## Supplementary Materials

Supplementary materials can be found at http://www.mdpi.com/1422-0067/19/10/3085/s1.
