# Supplementary material for: Specific Targeting of Plant and Apicomplexa Parasite Tubulin through Differential Screening Using In Silico and Assay-Based Approaches
Source: Int J Mol Sci. 2018 Oct 9;19(10):3085. doi: 10.3390/ijms19103085 (PMC6213459; doi:10.3390/ijms19103085)
Supplement: Supplementary file 1 [file ijms-19-03085-s001.pdf]

Supplementary

```
            1                                                              60
   TBA_PIG  MRECISIHVG QAGVQIGNAC WELYCLEHGI QPDGQMPSDK TIGGGDDSFN TFFSETGAGK
TBA1_BOVIN  MRECISVHVG QAGVQMGNAC WELYCLEHGI QPDGQMPSDK TIGGGDDSFT TFFCETGAGK
TBA1_ARATH  MREIISIHIG QAGIQVGNSC WELYCLEHGI QPDGTMPSDS TVGACHDAFN TFFSETSSGQ
 TBA_PLAFK  MREVISIHVG QAGIQVGNAC WELFCLEHGI QPDGQMPSDK ASRANDDAFN TFFSETGAGK

            61                                                            120
   TBA_PIG  HVPRAVFVDL EPTVIDEVRT GTYRQLFHPE QLITGKEDAA NNYARGHYTI GKEIIDLVLD
TBA1_BOVIN  HVPRAVFVDL EPTVIDEIRN GPYRQLFHPE QLITGKEDAA NNYARGHYTI GKEIIDPVLD
TBA1_ARATH  HVPRAVFLDL EPTVIDEVRT GTYRQLFHPE QLISGKEDAA NNFARGHYTV GREIVDTCLE
 TBA_PLAFK  HVPRCVFVDL EPTVVDEVRT GTYRQLFHPE QLISGKEDAA NNFARGHYTI GKEVIDVCLD

            121                                                           180
   TBA_PIG  RIRKLADQCT GLQGFSVFHS FGGGTGSGFT SLLMERLSVD YGKKSKLEFS IYPAPQVSTA
TBA1_BOVIN  RIRKLSDQCT GLQGFLVFHS FGGGTGSGFT SLLMERLSVD YGKKSKLEFS IYPAPQVSTA
TBA1_ARATH  RLRKLADNCT GLQGFLVFNA VGGGTGSGLG SLLLERLSVD FGKKSKLGFT IYPSPQVSTA
 TBA_PLAFK  RIRKLADNCT GLQGFLMFSA VGGGTGSGFG CLMLERLSVD YGKKSKLNFC CWPSPQVSTA

            181                                                           240
   TBA_PIG  VVEPYNSILT THTTLEHSDC AFMVDNEAIY DICRRNLDIE RPTYTNLNRL IGQIVSSITA
TBA1_BOVIN  VVEPYNSILT THTTLEHSDC AFMVDNEAIY DICRRNLDIE RPTYTNLNRL ISQIVSSITA
TBA1_ARATH  VVEPYNSVLS THSLLEHTDV VVLLDNEAIY DICRRSLDIE RPTYSNLNRL ISQTISSLTT
 TBA_PLAFK  VVEPYNSVLS THSLLEHTDV AIMLDNEAIY DICRRNLDIE RPTYTNLNRL IAQVISSLTA

            241                                                           300
   TBA_PIG  SLRFDGALNV DLTEFQTNLV PYPRAHFPLA TYAPVISAEK AYHEQLSVAE ITNACFEPAN
TBA1_BOVIN  SLRFDGALNV DLTEFQTNLV PYPRIHFPLA TYAPVISAEK AYHEQLSVAE ITNACFEPAN
TBA1_ARATH  SLRFDGAINV DITEFQTNLV PYPRIHFMLS SYAPVISSAK AYHEQFSVPE ITTSVFEPSN
 TBA_PLAFK  SLRFDGALNV DVTEFQTNLV PYPRIHFMLS SYAPVVSAEK AYHEQLSVSE ITNSAFEPAN

            301                                                           360
   TBA_PIG  QMVKCDPRHG KYMACCLLYR GDVVPKDVNA AIATIKTKRT IQFVDWCPTG FKVGINYEPP
TBA1_BOVIN  QMVKCDPRHG KYMACCLLYR GDVVPKDVNA AIAAIKTKRS IQFVDWCPTG FKVGINYQPP
TBA1_ARATH  MMAKCDPRHG KYMACCLMYR GDVVPKDVNT AVAAIKAKRT IQFVDWCPTG FKCGINYQPP
 TBA_PLAFK  MMAKCDPRHG KYMACCLMYR GDVVPKDVNA AVATIKTKRT IQFVDWCPTG FKCGINYQPP

            361                                                           420
   TBA_PIG  TVVPGGDLAK VQRAVCMLSN TTAIAEAWAR LDHKFDLMYA KRAFVHWYVG EGMEEGEFSE
TBA1_BOVIN  TVVPGGDLAK VQRAVCMLSN TTAIAEAWAR LDHKFDLMYA KRAFVHWYVG EGMEEGEFSE
TBA1_ARATH  SVVPGGDLAK VQRAVCMISN NTAVAEVFSR IDHKFDLMYS KRAFVHWYVG EGMEEGEFSE
 TBA_PLAFK  TVVPGGDLAK VMRAVCMISN STAIAEVFSR MDQKFDLMYA KRAFVHWYVG EGMEEGEFSE

            421                            453
   TBA_PIG  AREDMAALEK DYEEVGVDSV EGEGEEEGEE Y
TBA1_BOVIN  AREDMAALEK DYEEVGIDSY EDE--DEGEE
TBA1_ARATH  AREDLAALEK DYEEVGGEGA E-DDDEEGDE Y
 TBA_PLAFK  AREDLAALEK DYEEVGIESN EAEGEDEGYE ADY
```

Supplementary Figure 1

Supplementary Figure 1: Alignment of the sequences of *Bos taurus*, *Sus scrofa*, *Arabidopsis thaliana* and *Plasmodium falciparum* α-tubulin. Alignments have been performed using Multalin software with default options [1]. Highly conserved residues appear in red, weakly conserved in blue and divergent residues in black. High consensus regions predominate low ones indicating that α-tubulin is a highly conserved protein. Some plant/mammal differences (framed residues) are also detected in *P. falciparum* α-tubulin. The residues Ser6, Ile235 or Leu167 used in docking as criteria for the selection of ligands are highlighted in white on red background.

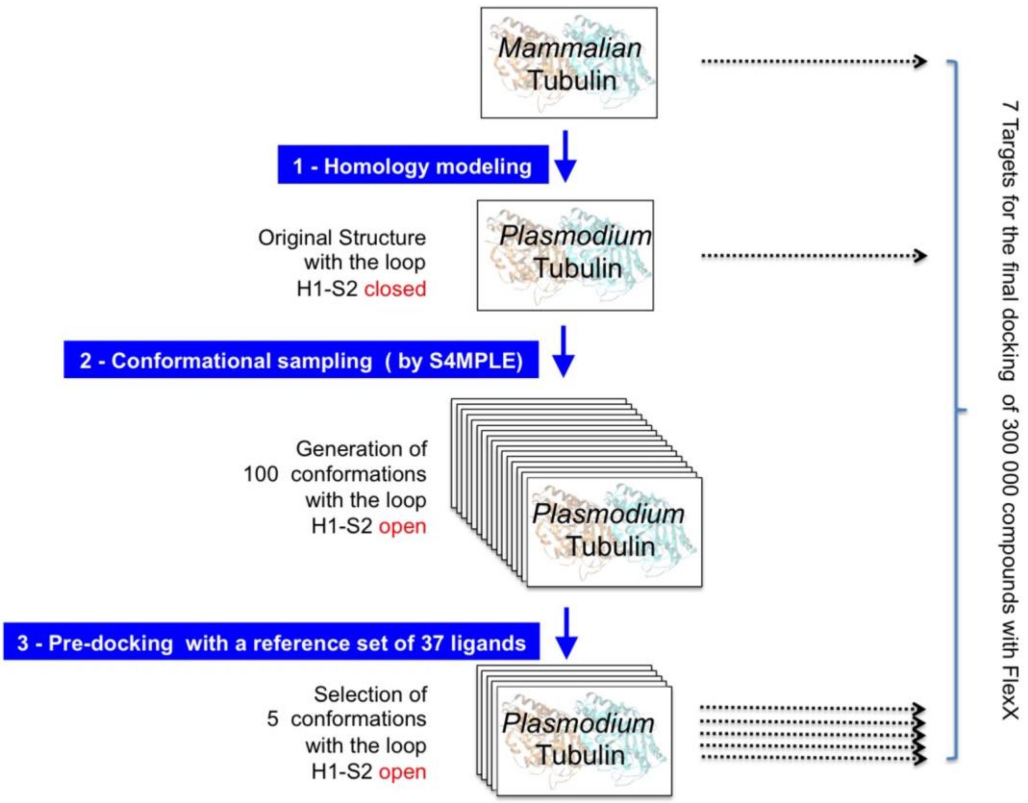

Supplementary Figure 2

Supplementary Figure 2 Strategy for the selection of potential 3D structures of plasmodium tubulin to be used as targets for a virtual screen of compounds with FlexX. First, the *Plasmodium* tubulin 3D structure has been predicted by homology with the *Mammalian* tubulin. This original structure has a loop (H1-S2 loop) that locks the dinitroanaline binding site. Using S4MPLE [2], a conformational sampler software, 100 **open** conformations of this loop were generated. A set of 37 molecules (10 known to bind to the dinitroaniline binding site and 27 not having been described to bind tubulin) was docked against the dinitroaniline binding site of these 100 conformations. The 5 conformations which best discriminated the active molecules from the inactive ones were selected. These 5 conformations, the mammalian tubulin and the original *Plasmodium* tubulin conformations were the final targets for the docking of 300 000 compounds with FlexX in the dinitroaniline binding site.

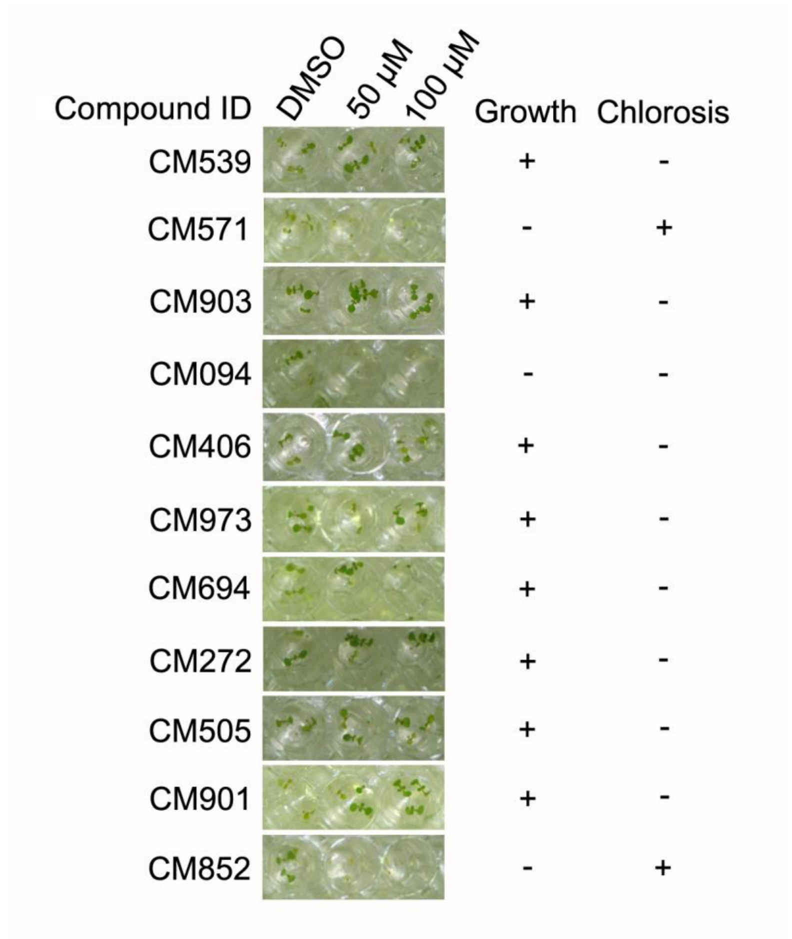

Supplementary Figure 3

Supplementary Figure 3: Effect of the selected compounds on *Arabidopsis thaliana* growth
Arabidopsis seedlings were sawn on 24-well microplates containing an agar-based solid medium containing compounds, as indicated. After 7 days of incubation in a growth chamber, growth was monitored.

Supplementary references

1. Corpet F: Multiple sequence alignment with hierarchical clustering. *Nucleic Acids Res.* 1988, 16:10881–90.
2. Hoffer L, Chira C, Marcou G, Varnek A, Horvath D: S4MPLE--Sampler for Multiple Protein-Ligand Entities: Methodology and Rigid-Site Docking Benchmarking.. *Molecules* 2015, 20:8997–9028.
